# Automatic Estimation of Multidimensional Personality From a Single Sound-Symbolic Word

**DOI:** 10.3389/fpsyg.2021.595986

**Published:** 2021-04-22

**Authors:** Maki Sakamoto, Junji Watanabe, Koichi Yamagata

**Affiliations:** ^1^Department of Informatics, The University of Electro-Communications, Chofu, Japan; ^2^NTT Communication Science Laboratories, Nippon Telegraph and Telephone Corporation, Atsugi, Japan

**Keywords:** personality evaluation, system construction, sound-symbolic word, onomatopoeia, Japanese

## Abstract

Researchers typically use the “big five” traits (Extroversion, Agreeableness, Conscientiousness, Neuroticism, and Openness) as a standard way to describe personality. Evaluation of personality is generally conducted using self-report questionnaires that require participants to respond to a large number of test items. To minimize the burden on participants, this paper proposes an alternative method of estimating multidimensional personality traits from only a single word. We constructed a system that can convert a sound-symbolic word (SSW) that intuitively expresses personality traits into information expressed by 50 personality-related adjective pairs. This system can obtain information equivalent to the adjective scales using only a single word instead of asking many direct questions. To achieve this, we focused on SSWs in Japanese that have the association between linguistic sounds and meanings and express diverse and complex aspects of personality traits. We evaluated the prediction accuracy of the system and found that the multiple correlation coefficients for 48 personality-related adjective pairs exceeded 0.75, indicating that the model could explain more than half of the variations in the data. In addition, we conducted an evaluation experiment in which participants rated the appropriateness of the system output using a seven-point scale (with −3 as absolutely inappropriate and +3 as completely appropriate). The average score for 50 personality-related adjective pairs was 1.25. Thus, we believe that this system can contribute to the field of personality computing, particularly in terms of personality evaluation and communication.

## Introduction

Understanding personality is crucial in making sense of our relationships with others. Most evaluations of personality traits, such as the “big five,” use questionnaires as self-report measures. However, these questionnaires generally ask people to respond to a large number of test items with two extreme positions or polar opposites described using adjectives. To ease the burden on participants, in this paper, we introduce a novel method for evaluating personality traits using only a single word. Specifically, we constructed a system that can convert a word that intuitively expresses personality traits into information equivalent to evaluations derived from 50 pairs of adjectives for big five personality traits. In other words, this system can generate the information of 50

adjectival scales from only a single word, instead of asking participants a lengthy series of questions. Our system is thus an efficient method for reducing the burden on people expressing their own personality, and therefore has applications to the field of personality computing that deal with personality evaluation and communication (see Vinciarelli and Mohammad, 2014 for a review).

The evaluation of the “big five” personality traits has a long history (Allport and Odbert, 1936; Cattell, 1943; Fiske, 1949; Tupes and Christal, 1961; Norman, 1967). In a landmark study, Goldberg (1982) extracted five factors; Extroversion, Agreeableness, Conscientiousness, Neuroticism, and Openness, and McCrae and Costa (1987) showed that these five factors could be reliably extracted regardless of whether the data were collected using self-rating or rating by others. The big five personality traits have since been evaluated using self-report questionnaires or the completion of questionnaires by others in studies conducted in a wide range of languages including English, German, Dutch, Czech, Polish, Russian, Italian, Spanish, Hebrew, Hungarian, Turkish, Korean, Tagalog, and Japanese. Word-related personality studies are still frequently conducted worldwide. Since recent studies have identified systematic associations between personality and language use (Pennebaker and King, 1999; Hirsh and Peterson, 2009), there are a large-scale personality analyses using the standard word-based categories provided in the Linguistic Inquiry and Word Count (LIWC) 2001 program (Pennebaker et al., 2001). LIWC is the most commonly used language analysis program in studies investigating the relation between word use and psychological variables. Yarkoni (2010) analyzed personality and word use among bloggers focusing 66 LIWC categories. Das and Das (2016) developed a lexicon of words and phrases corresponding to each of the big five personality classes using LIWC text analysis tool (Mairesse et al., 2007), which categorizes words into psychologically meaningful categories, including personality-related groups.

**FIGURE 1:**
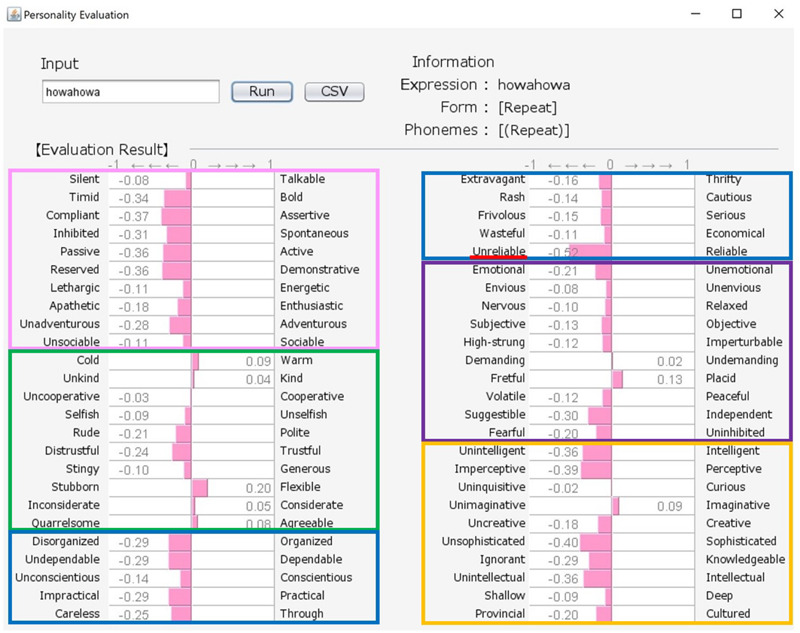
Output for “howa-howa (nearly equal to fluffy)” personality (soft but unreliable). The pink, green, blue, purple, and orange frames indicate Extroversion, Agreeableness, Conscientiousness, Neuroticism, and Openness, respectively.

**FIGURE 2:**
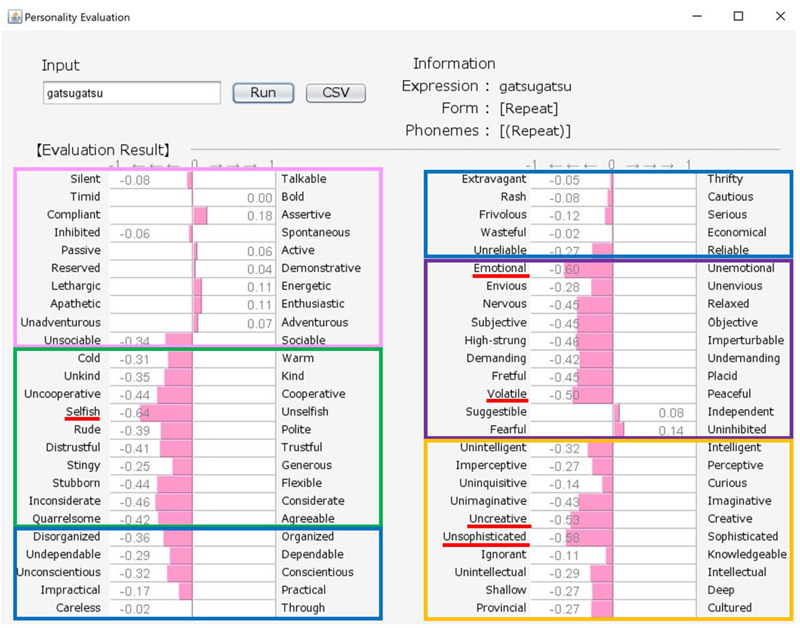
Output for “gatsu-gatsu (greedy)” personality (aggressive and unpleasantly positive). The pink, green, blue, purple, and orange frames indicate Extroversion, Agreeableness, Conscientiousness, Neuroticism, and Openness, respectively.

Although there exist many reliable and widespread measures of personality, these are often questionnaires containing many adjectives, and can represent a burden on participants if they are asked to respond to a large number of test items. Here, we propose an engineering solution to this problem by building a new system that uses a word class referred to as “sound-symbolic words” (hereafter, SSWs). In the system, a SSW that can intuitively express personality traits is inputted, and evaluations against 50 personality-related adjective pairs are then produced based on analyses of the sounds of the inputted SSW (see samples of system output in Figures 1, 2). SSWs are adjective-like words that have associations between their sound and meaning. The existence of SSWs has been demonstrated in a wide variety of languages (e.g., Japanese, Chinese, Korean, Indonesian, Finnish, English, French, German, Modern Greek, and Native languages in North America, Latin America, Asia, Australia, and Africa).

There are two main reasons for our focus on SSWs. First, SSWs can describe a complex personality using a single word. For example, Japanese people use not only adjectives but also SSWs (called “onomatopoeia” in Japanese) that can describe complicated personality traits with a single word. “Honwaka” (a SSW in Japanese) means an agreeable, friendly, and calm personality. A single SSW in Japanese tends to convey information that is more diverse and complex than what can be expressed by one adjective. Uchida (2005) reported that SSWs can indicate changes in impressions of personality that the big five cannot adequately describe, and Nishioka et al. (2006) suggested that SSWs can express aspects of personality that do not easily fit into the big five framework. There are many SSWs in Japanese. Komatsu et al. (2012) extracted about 120 personality-related SSWs from the Kojien word dictionary (5th edition), and extracted 60 SSWs in the format of a personality test.

Second, we focused on SSWs because they have systematic sound-symbolic features (Jespersen, 1922; Köhler, 1929; Sapir, 1929, for early studies; Hamano, 1998 for Japanese SSWs). For instance, the phonemes of Japanese SSWs, especially initial consonants, are known to characterize categories of touch. The two words “sara-sara” (smooth, dry, and comfortable) and “zara-zara” (rough, hard, and uncomfortable) differ only in their initial sounds (/s/ or /z/). This difference in only one feature can convey a critical difference in the perception and evaluation of texture (e.g., Sakamoto and Watanabe, 2018). In a previous study (Doizaki et al., 2017), we used this sound-symbolic feature to evaluate complex sensations of touch, and developed a method for calculating the multidimensional ratings of a word by integrating the impressions of each phoneme. This system can convert a SSW in Japanese into quantitative ratings in multiple tactile dimensions. Another study also quantified the impressions of SSWs and used for description of robot motion (Ito et al., 2013).

In terms of information related to personality, sound-symbolic features can also be observed. The sounds of certain Japanese words are associated with features of personality traits (Shinohara and Kawahara, 2013). Lindauer (1990) demonstrated that English speakers judged the sound “takete” to be unfriendly and tough, whereas the sound “maluma” was considered to be friendly and tender. Milán et al. (2013) reported that Spanish speakers judged the word “kiki” to be clever and nervous. Kawahara et al. (2015) showed that Japanese and English speakers associate similar sound-symbolic features with personality traits: “difficult” personalities are typically associated with a phonetic class of sounds called obstruents. Thus, it appears that SSWs can be used as clues to evaluate and analyze personality traits based on their phonetic features. For these two reasons, we hypothesized that multidimensional ratings of personality could be predicted by combining the evaluations of each phoneme in a SSW, and that this could be advantageous for our system. In the next section, we describe the construction and evaluation of the proposed system in detail.

## System Construction

We constructed a database that includes the sound-symbolic associations of phonemes and associated ratings of personality traits. In the experiment, participants viewed Japanese SSWs displayed on a monitor and rated their impressions of these words in terms of the 50 polar opposite adjective scales. From the results, we obtained a quantitative rating database for each phoneme of the SSWs, which enabled us to estimate impressions of a word by analyzing only the phonemes of the word. This database is the foundation for automatic conversion of Japanese SSWs into values related to the 50 pairs of adjectives of personality traits.

### Participants

Thirty-two paid participants, aged 20–27 years (18 men and 14 women), participated in this experiment. They had no linguistics knowledge and were all native Japanese speakers. They had normal or corrected-to-normal vision, and none of them reported any visual or linguistic impairments. They were unaware of the purpose of the experiment, and informed consent was obtained from all the participants before the experiment started. All of the experiments, including those for system evaluation, were performed at the University of Electro-Communications, Japan. Experimental procedures were conducted in accordance with the ethical standards outlined by the Declaration of Helsinki.

### Stimuli

To obtain sound-symbolic associations of all Japanese phonemes with the 50 pairs of adjectives of the big five, we selected word stimuli that included all varieties of Japanese phonemes. First, we decided to use all 60 SSWs given in Komatsu et al. (2012). Then, we added an additional 66 SSWs to cover all Japanese phonemes. The total number of SSWs used in the experiment was 126, as shown in Table 1. Each SSW was evaluated by ten participants. That is, the 126 SSWs were divided into 5 groups (25 or 26 words each), and 18 of the 32 participants evaluated 2 different groups, while 14 participants evaluated 1 group.

**TABLE 1:** All 126 sound-symbolic words (SSWs) used in the experiment (SSWs used in the study by Komatsu are shown with *).

1	ODO-ODO*	27	KEROQ*	53	SHIME-SHIME	79	GUNYAHRI	105	SABA-SABA*
2	SARARI*	28	FUNI-FUNI	54	OQTORI*	80	YUHRURI	106	SUI-SUI
3	HENA-HENA	29	HONWAKA*	55	UKI-UKI*	81	GIH-GIH	107	SARAQ*
4	NONBIRI*	30	CHARA-CHARA*	56	AWA-AWA	82	JIME-JIME*	108	KUNE-KUNE
5	DERE-DERE*	31	ZAWA-ZAWA	57	NUME-NUME	83	DARUN-DARUN	109	KII-KII
6	MOWA-MOWA	32	SHINA-SHINA	58	KIRIQ*	84	NIYAKERU*	110	HOWAHRI*
7	FUNWARI	33	KYAH-KYAH*	59	IRA-IRA*	85	SHIQKARI*	111	CHANTO*
8	SHEKA-SHEKA	34	WAKYA-WAKYA	60	NOHOHON*	86	YOWA-YOWA	112	BOKEQ*
9	SHAKIQ*	35	MOJI-MOJI*	61	DOSHIN	87	HONYA-HONYA	113	YORO-YORO
10	KUYO-KUYO*	36	KARAQ*	62	ORO-ORO*	88	TOGE-TOGE*	114	BUSUQ*
11	GONERU*	37	WAI-WAI	63	UKYA-UKYA	89	SUQKIRI*	115	KERORI*
12	FUHWA-FUHWA	38	NUPU-NUPU	64	NECHIKOI*	90	DYURU-DYURU	116	GITYU-GITYU
13	KYAPI-KYAPI	39	GAMI-GAMI*	65	DAHRA-DAHRA	91	MUSUQ*	117	SAQPARI*
14	KARI-KARI*	40	GUHTARA	66	CHIMI-CHIM	92	BEQTARI*	118	FOWA-FOWA
15	FUHWARI	41	MOMA-MOMA	67	WAKI-WAKI	93	BONYARI*	119	ZUSHIN
16	CHIMA-CHIMA	42	WARA-WARA	68	BOUQ*	94	KICHIN*	120	NIKO-NIKO*
17	GASATSU*	43	KEBAI*	69	POKA-POKA	95	FUWA-FUWA*	121	SOWA-SOWA
18	POWA-POWA	44	FUNYA-FUNYA	70	RUN-RUN	96	YURU-YURU	122	CHARANPORAN*
19	KOSHO-KOSHO	45	YORE-YORE	71	SARAHRI	97	KAQ*	123	POWAN
20	YOTA-YOTA	46	SUPAQ	72	PEKO-PEKO	98	SUKAQ*	124	PERA-PERA*
21	WACHA-WACHA	47	PURA-PURA	73	ASHE-ASHE	99	KUTYI-KUTYI	125	MAQTARI*
22	PARIQ	48	GUFO-GUFO	74	BOSO-BOSO*	100	PIRI-PIRI*	126	KICHIQ*
23	FASA-FASA	49	MESO-MESO*	75	GATSUN	101	KUFO-KUFO		
24	NAYO-NAYO*	50	FUWAHN	76	RIN-RIN	102	FUWAN		
25	BISHIQ*	51	UJI-UJI*	77	KIQCHIRI*	103	AQSARI*		
26	BIKU-BIKU*	52	BETA-BETA*	78	RAN-RAN	104	SHIO-SHIO		

The participant sat in front of a 23-inch LCD monitor. The SSWs were presented on the monitor with a resolution of 1024 × 768 pixels and a refresh rate of 60 Hz. The SSWs were presented in 11-pt MS PGothic font. The distance between the participant’s eyes and the screen was approximately 50 cm. The polar opposite scales were presented in the form of an answer matrix on the monitor. The rating scales, shown in Table 2, included 50 pairs of adjectives used for expressing personality traits of the big five. Each participant responded to all 50 rating scales for each SSW.

**TABLE 2:** Fifty rating scales for evaluating personality, with examples of values for “howa”.

Big Five	Rating scales	Consonants		Vowels		R	R_SSW_
		/h/ (X_1_)	/w/ (X_7_)	/o/ (X_4_)	/a/ (X_10_)		
Extroversion	Silent – Talkable	−0.074	0.025	−0.156	0.114	0.601	0.807
	Timid – Bold	−0.048	−0.231	−0.100	0.073	0.622	0.814
	Compliant – Assertive	−0.063	−0.237	−0.096	0.011	0.617	0.823
	Inhibited – Spontaneous	−0.112	−0.080	−0.135	0.029	0.643	0.822
	Passive – Active	−0.132	−0.119	−0.154	0.050	0.644	0.818
	Reserved – Demonstrative	−0.096	−0.145	−0.147	0.074	0.622	0.839
	Lethargic – Energetic	−0.108	0.016	−0.104	0.040	0.616	0.812
	Apathetic – Enthusiastic	−0.120	0.084	−0.126	0.000	0.523	0.767
	Unadventurous – Adventurous	−0.050	−0.192	−0.107	0.084	0.596	0.804
	Unsociable – Sociable	0.037	−0.018	−0.115	−0.022	0.563	0.778

Agreeableness	Cold – Warm	0.029	0.050	−0.005	0.012	0.527	0.778
	Unkind – Kind	0.046	0.021	−0.003	−0.013	0.541	0.779
	Uncooperative – Cooperative	0.044	0.021	−0.018	−0.012	0.544	0.767
	Selfish – Unselfish	0.061	0.046	0.023	0.012	0.495	0.791
	Rude – Polite	−0.007	0.030	0.001	−0.090	0.610	0.826
	Distrustful – Trustful	0.017	−0.002	−0.023	−0.068	0.584	0.793
	Stingy – Generous	0.013	−0.147	−0.031	0.064	0.519	0.778
	Stubborn – Flexible	0.069	0.019	−0.040	0.040	0.539	0.800
	Inconsiderate – Considerate	0.072	0.040	−0.005	−0.048	0.545	0.801
	Quarrelsome – Agreeable	0.027	−0.019	0.033	0.004	0.580	0.806

Conscientiousness	Disorganized – Organized	0.075	−0.134	−0.047	−0.062	0.528	0.826
	Undependable – Dependable	−0.017	−0.059	−0.019	−0.087	0.602	0.796
	Unconscientious – Conscientious	0.033	−0.039	0.021	−0.060	0.582	0.804
	Impractical – Practical	0.015	−0.065	−0.044	−0.064	0.562	0.805
	Careless – Through	−0.042	0.036	−0.020	−0.106	0.499	0.780
	Extravagant – Thrifty	0.017	0.038	−0.010	−0.125	0.505	0.812
	Rash – Cautious	−0.057	0.153	0.010	−0.138	0.494	0.827
	Frivolous – Serious	0.007	0.013	0.030	−0.141	0.579	0.817
	Wasteful – Economical	0.061	0.025	0.017	−0.113	0.509	0.824
	Unreliable – Reliable	−0.061	−0.181	−0.028	−0.034	0.608	0.792

Neuroticism	Emotional – Unemotional	0.061	−0.210	0.084	−0.001	0.562	0.819
	Envious – Unenvious	0.000	−0.214	0.068	0.017	0.579	0.810
	Nervous – Relaxed	0.019	−0.150	0.031	0.072	0.582	0.776
	Subjective – Objective	0.021	−0.076	0.033	0.018	0.500	0.822
	High-strung – Imperturbable	0.023	−0.240	0.045	0.056	0.550	0.789
	Demanding – Undemanding	0.019	−0.016	0.057	0.008	0.553	0.802
	Fretful – Placid	0.049	−0.045	0.076	0.013	0.593	0.799
	Volatile – Peaceful	0.127	−0.165	0.059	−0.037	0.536	0.825
	Suggestible – Independent	−0.020	−0.256	−0.110	0.026	0.542	0.816
	Fearful – Uninhibited	−0.047	−0.255	−0.103	0.114	0.537	0.795

Openness	Unintelligent – Intelligent	−0.045	−0.162	−0.074	−0.011	0.539	0.784
	Imperceptive – Perceptive	0.003	−0.126	−0.163	−0.038	0.555	0.811
	Uninquisitive – Curious	−0.074	0.065	−0.065	−0.014	0.444	0.736
	Unimaginative – Imaginative	0.008	0.095	0.030	−0.019	0.435	0.718
	Uncreative – Creative	−0.038	0.049	−0.026	−0.017	0.442	0.784
	Unsophisticated – Sophisticated	0.000	−0.014	−0.084	−0.088	0.597	0.839
	Ignorant – Knowledgeable	−0.010	0.019	−0.044	−0.084	0.496	0.775
	Unintellectual – Intellectual	−0.008	0.015	−0.018	−0.096	0.549	0.806
	Shallow – Deep	0.005	0.128	0.055	−0.085	0.452	0.793
	Provincial – Cultured	−0.023	0.037	−0.013	−0.067	0.526	0.770

### Procedure

The trials started with the presentation of a SSW on the monitor. The participants were asked to report how they felt about each word on a seven-point SD scale, e.g., for the silent–talkable scale, participants selected one of the following seven points: **−**3, very silent; **−**2, silent; **−**1, slightly silent; 0, neither; +1, slightly talkable; +2, talkable; and, +3, very talkable. The participants responded by pushing one of seven buttons. The time allotted for answering was unlimited, but most participants took less than 1 min per trial. The presentation order of the SSWs was randomized among participants. The order and polarity of the scales were also randomized in the answer matrix.

### Personality Estimation Model

The experimental results produced 63,000 datapoints (50 rating scales × 126 words × 10 participants). To estimate the impressions of SSWs, we created a linear regression model in which the following equation was used to predict each rating value:

(1) $$Y=\sum_{i=1}^{13}X_{i}+Const.$$

where *Y* represents the rating values of the respective 50 scales, and *X*_1_ – *X*_13_ are quantified values of phonemes. *X*_1_ – *X*_6_, respectively are the values of the specific consonant, voiced sound/p-sound, contracted sounds, vowels, semivowels, and special phonemes in the first syllable. *X*_7_ – *X*_12_ represent the same categories for the second syllable, respectively, and *X*_13_ denotes the presence or absence of repetitions in the word (see also Table 3). Using the rating values as the objective variables and the variation of phonemes as the predictor variables, we conducted mathematical quantification theory class I, which is a type of multiple regression analysis.

**TABLE 3:** Correspondence between variables and phonemes.

First syllable	Second syllable	Phonological characteristics	Variation of phonemes
*X*_1_	*X*_7_	Consonants	/k/, /s/, /t/, /n/, /h/,/m/, /y/, /r/, /w/ or absence
*X*_2_	*X*_8_	Voiced sounds, /p/-sounds	Presence (/g/, /z/, /d/, /b/, /p/) or absence
*X*_3_	*X*_9_	Contracted sounds	Presence(/ky/, /sy/, /ty/, /ny/, /hy/, /my/, /ry/, /gy/, /zy/, /by/, /py/) or absence
*X*_4_	*X*_10_	Vowels	/a/, /i/, /u/, /e/, /o/
*X*_5_	*X*_11_	Semi-vowels	/a/, /i/, /u/, /e/, /o/ or absence
*X*_6_	*X*_12_	Special sounds	/N/, /Q/, /R/, /Li/ or absence
*X*_13_		Repetition	Presence(ex. huwa-huwa) or absence

Table 2 shows examples of the results of the SSW “howa.” According to Equation (1), the rating values of a SSW can be calculated by the linear sum of the values (*X*_1_ – *X*_13_) of the word. The expression “howa” is composed of the first mora /ho/ (/h/ + /o/) and the second mora /wa/ (/w/ + /a/). Therefore, the value of the unreliable–reliable scale on a seven-point scale (unreliable **−**3 to reliable 3) divided by three is estimated by the following equation [see Equation (1) and Table 2]. The estimated value of −0.304 suggests that “howa” is associated with an unreliable personality.

Y=/h/+/o/+/w/+/a/+Cosnt.

$$=/h/\left(X_{1}\right)+\mathrm{absence}\left(X_{2}\right)+\mathrm{absence}\left(X_{3}\right)+/o/\left(X_{4}\right)+\mathrm{absence}\left(X_{5}\right)+\mathrm{absence}\left(X_{6}\right)+/w/\left(X_{7}\right)+\mathrm{absence}\left(X_{8}\right)+\mathrm{absence}\left(X_{9}\right)+/a/\left(X_{10}\right)+\mathrm{absence}\left(X_{11}\right)+\mathrm{absence}\left(X_{12}\right)+\mathrm{absence}\left(X_{13}\right)+\mathrm{Const}.$$

=(-0.061)+(0.079)+(0.02)+(-0.028)+(-0.005)+(-0.042)+(-0.181)+(0.035)+(0.007)+(-0.034)+(0.013)+(-0.033)+(0.133)+(-0.207)=-0.304

The multiple correlation coefficient, R, shown in Table 2 is calculated by

(2) $$R^{2}=1-\frac{RSS}{TSS}=1-\frac{RSS}{ESS+RSS},$$

where RSS is the residual sum of squares, and TSS is the total sum of squares that can be partitioned into the explained sum of squares (ESS) and the RSS, i.e.,

(3) TSS=ESS+RSS,

due to the Pythagorean equation in the sample space *ℝ*^n^ with *n* = 1260. Because ten participants in the experiment evaluated each SSW, each RSS could be further partitioned into the sum of squares for the residual of participant differences, *RSS_p*, and the residual of SSW differences, RSS_SSW_, i.e.,

(4) $$RSS=RSS_{p}+RSS_{SSW}.$$

Note that averaging the ratings of the 10 participants can be regarded as an orthogonal projection in the sample space *ℝ*^n^, as in the linear regression. Thus, the Pythagorean Equations (3) and (4) are possible. The multiple correlation coefficients without participant differences, *R*_*SSW*_, can be calculated by

(5) $$R_{SSW}^{2}=1-\frac{RSS_{SSW}}{ESS+RSS_{SSW}}.$$

The multiple correlation coefficients, *R*_*SSW*_, were used as indicators of prediction accuracy. We can see that all ten multiple values of *R*_*SSW*_ for Extroversion, Agreeableness, Conscientiousness, Neuroticism, and 8 of the 10 for Openness exceeded 0.75, indicating that the model explains more than half of variations in the data.

### User Interface and Information Processing

Our system comprises a user interface module, a SSW-parsing module, an analyzing module, and a database. Figures 1, 2 show the system’s estimation values for “howa-howa (nearly equal to fluffy)” and “gatsu-gatsu (greedy).” When a user inputs a SSW into the text field in the upper-left frame of a window and presses the “Run” button, the parsing module automatically divides the word into each phoneme and classifies its form. On the basis of (1) and the database, the analyzing module calculates the rating values of the word for the 50 scales. Then, the module converts the calculated values from −3 to 3 into values from −1 to 1. Finally, graphs of the estimated values of the word are displayed in the lower frame. The form and phonemic elements of the word are displayed in the upper-right frame.

## System Evaluation

Here we describe an experiment we conducted to verify the validity of the system constructed in this study.

### Participants

Seven participants aged 22–27 years (4 men and 3 women) participated in the evaluation experiment. The participants were familiar with each other so that each participant could evaluate the personality of the target person on the basis of their friendship.

### Procedure

**FIGURE 3:**
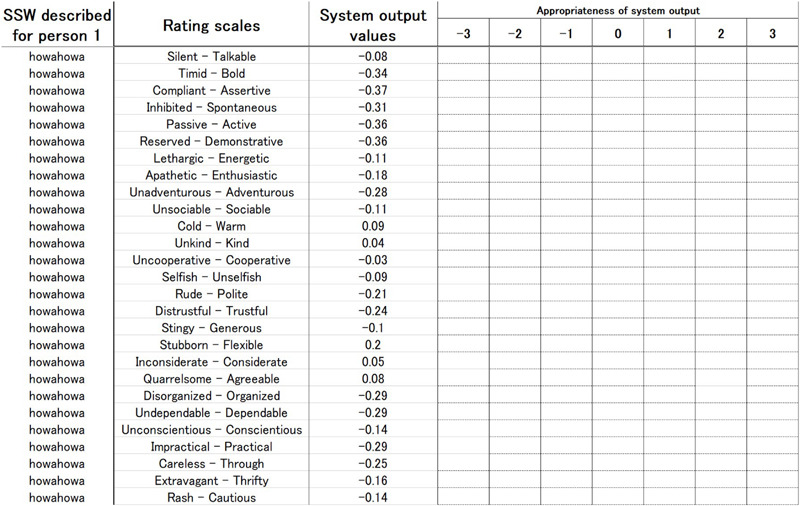
Example of evaluation form (SSW described for person X; rating scales; system output value; and appropriateness of system output).

Each of the seven participants spontaneously described the personality traits of the other six people using SSWs. Thus, a total of 42 SSWs were obtained. The 42 SSWs were then analyzed by the system constructed in this study, and values of the 50 scales were obtained. Each participant also rated the output values of the 50 scales for each of the other six persons from −3 as “absolutely inappropriate” to +3 as “completely appropriate.” Figure 3 shows an example of the evaluation form.

### Results

In this evaluation experiment, each scale was evaluated 42 times, which is sufficient for the central limit theorem to be applied. Therefore, the average scores followed normal distributions, and the *Z*-test was valid. Table 4 shows the average scores and *Z*-values of the *Z*-tests for each scale. Because the *Z*-values for all 50 scales exceeded 2, the average scores were significantly above zero at the 0.025 level, indicating that the participants tended to give positive evaluations to all the scales. The average scores for Extroversion, Agreeableness, Conscientiousness, Neuroticism, and Openness were 1.19, 1.34, 1.18, 1.53, and 1.02, respectively. The average Z-values for Extroversion, Agreeableness, Conscientiousness, Neuroticism, and Openness were 4.80, 5.26, 4.82, 7.28, and 4.16, respectively. Although the scores for Openness were slightly lower than the others, this tendency was similar to that observed for the multiple correlation coefficient (*R* and *R*_*SSW*_ in Table 2).

**TABLE 4:** Mean values and *Z*-values of evaluation values.

Big Five	Rating scales	Mean	*Z*	Big Five	Rating scales	Mean	*Z*
Extroversion	Silent – Talkable	1.07	3.77	Conscientiousness	Extravagant – Thrifty	1.17	4.17
	Timid – Bold	0.90	3.31		Rash – Cautious	1.14	4.35
	Compliant – Assertive	1.52	6.57		Frivolous – Serious	1.24	4.62
	Inhibited – Spontaneous	1.21	4.72		Wasteful – Economical	1.00	3.70
	Passive – Active	1.55	6.44		Unreliable – Reliable	0.88	2.92
	Reserved – Demonstrative	1.40	6.13	Neuroticism	Emotional – Unemotional	1.24	5.29
	Lethargic – Energetic	1.14	4.89		Envious – Unenvious	1.71	7.64
	Apathetic – Enthusiastic	0.98	3.74		Nervous – Relaxed	1.83	9.80
	Unadventurous – Adventurous	0.69	2.78		Subjective – Objective	1.60	8.36
	Unsociable – Sociable	1.38	5.70		High-strung – Imperturbable	1.40	6.05
Agreeableness	Cold – Warm	1.50	5.82		Demanding – Undemanding	1.24	5.43
	Unkind – Kind	1.26	4.61		Fretful – Placid	1.55	6.57
	Uncooperative – Cooperative	1.38	5.17		Volatile – Peaceful	1.79	9.21
	Selfish – Unselfish	1.12	4.59		Suggestible – Independent	1.67	9.09
	Rude – Polite	1.26	3.98		Fearful – Uninhibited	1.29	5.36
	Distrustful – Trustful	1.10	3.81	Openness	Unintelligent – Intelligent	1.57	6.13
	Stingy – Generous	1.29	5.29		Imperceptive – Perceptive	1.64	6.90
	Stubborn – Flexible	1.69	8.30		Uninquisitive – Curious	1.26	5.04
	Inconsiderate – Considerate	1.31	4.96		Unimaginative – Imaginative	0.93	4.05
	Quarrelsome – Agreeable	1.50	6.04		Uncreative – Creative	0.67	2.66
Conscientiousness	Disorganized – Organized	1.81	11.61		Unsophisticated – Sophisticated	0.69	2.73
	Undependable – Dependable	0.88	2.72		Ignorant – Knowledgeable	0.83	3.36
	Unconscientious – Conscientious	1.12	3.72		Unintellectual – Intellectual	0.64	2.33
	Impractical – Practical	1.19	4.62		Shallow – Deep	0.81	3.50
	Careless – Through	1.40	5.78		Provincial – Cultured	1.17	4.86

## Discussion

Many studies of personality have been based on the “big five” hypothesis proposed by Goldberg (1982), which suggests that human personality is classified into five factors derived from 100 adjectives. A questionnaire method for evaluating personality using these five categories is well-established and widely used today. Costa and MacCrae (1992); Goldberg (1992)Goldberg (1993), and Goldberg et al. (2006) further developed the scale to measure personality based on big five personality traits. However, personality evaluations using lengthy questionnaires can burden the respondents. There are many online sites based on big five theory, which usually take around 10 min. The following sites are examples: https://openpsychometrics.org/tests/IPIP-BFFM accessed March 12, 2021, which is referring to Goldberg (1992), and https://www.123test.com/personality-test/, accessed February 28, 2021. In contrast, in our system, when a word that intuitively expresses personality is inputted into the text field, information equivalent to evaluations against multiple personality-related adjectives is instantly generated on the basis of analyses of the sounds in the word. All the participant has to do is provide only one word for an answer. We believe that our system represents an alternative method for evaluating personality.

The proposed system might function effectively not only for self-evaluation but also in situations where personality evaluation by others is required. For instance, it could be particularly valuable when one needs to understand someone’s personality with limited available information. For example, in a business situation, a salesperson may understand elements of a customer’s personality after only a brief description given by colleagues. In an educational setting, a new teacher could understand a student’s personality through descriptions given by other students. Our study expands personality research into the field of engineering application, and proposes a novel system for capturing complex personality traits.

**FIGURE 4:**
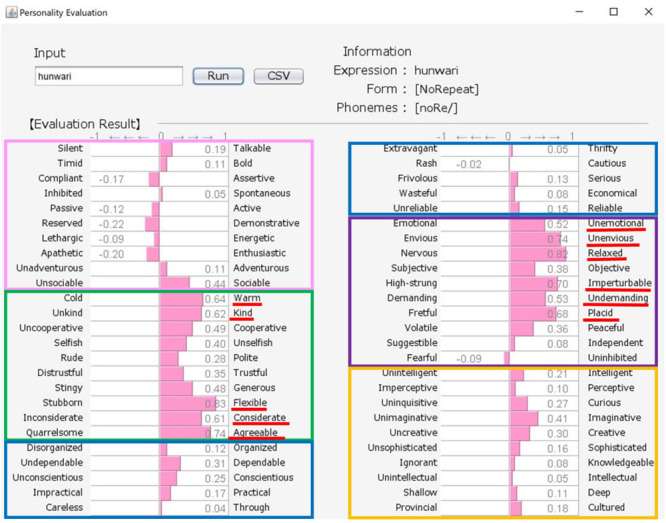
Output for “hunwari (gentle)” personality. The pink, green, blue, purple, and orange frames indicate Extroversion, Agreeableness, Conscientiousness, Neuroticism, and Openness, respectively.

**FIGURE 5:**
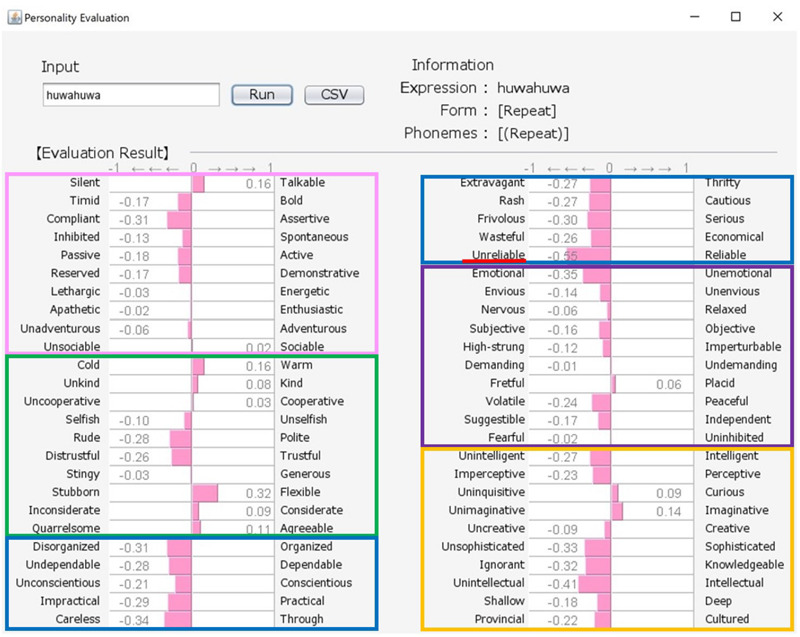
Output for “huwahuwa (nearly equal to fluffy)” personality. The pink, green, blue, purple, and orange frames indicate Extroversion, Agreeableness, Conscientiousness, Neuroticism, and Openness, respectively.

**FIGURE 6:**
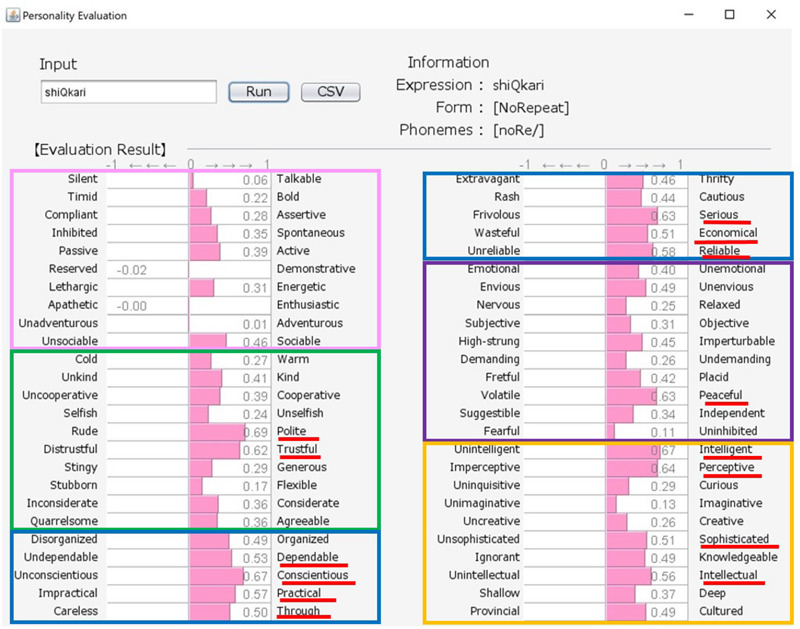
Output for “shikkari (solid)” personality. The pink, green, blue, purple, and orange frames indicate Extroversion, Agreeableness, Conscientiousness, Neuroticism, and Openness, respectively.

**FIGURE 7:**
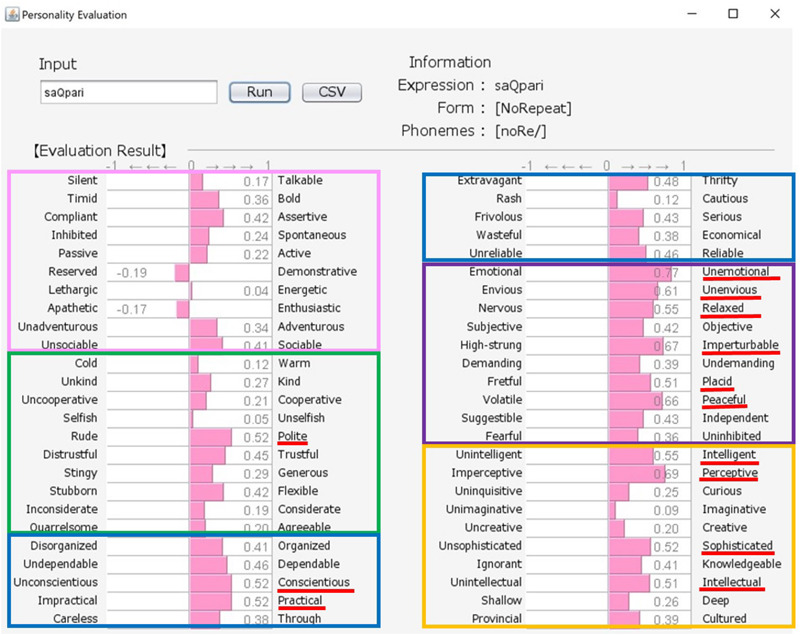
Output for “sappari (nearly equal to frank)” personality. The pink, green, blue, purple, and orange frames indicate Extroversion, Agreeableness, Conscientiousness, Neuroticism, and Openness, respectively.

**FIGURE 8:**
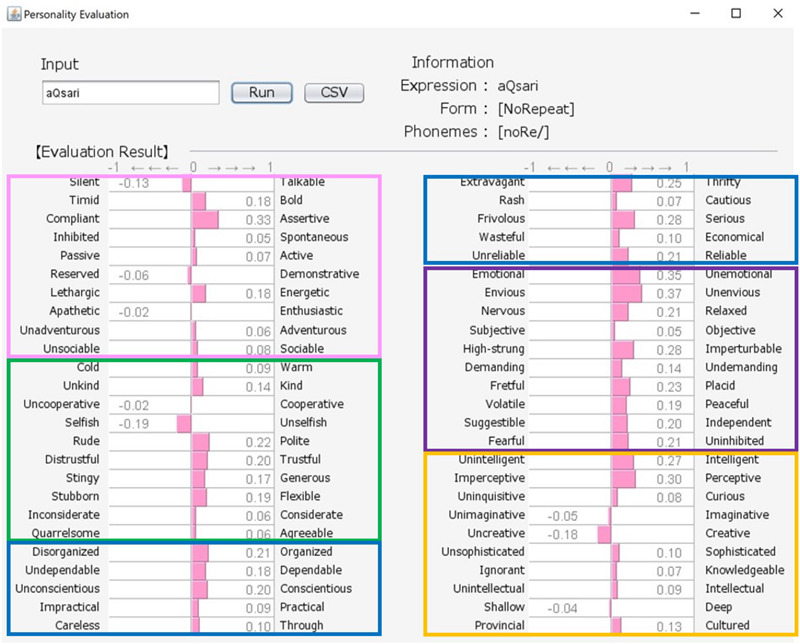
Output for “assari (standoffish)” personality. The pink, green, blue, purple, and orange frames indicate Extroversion, Agreeableness, Conscientiousness, Neuroticism, and Openness, respectively.

**FIGURE 9:**
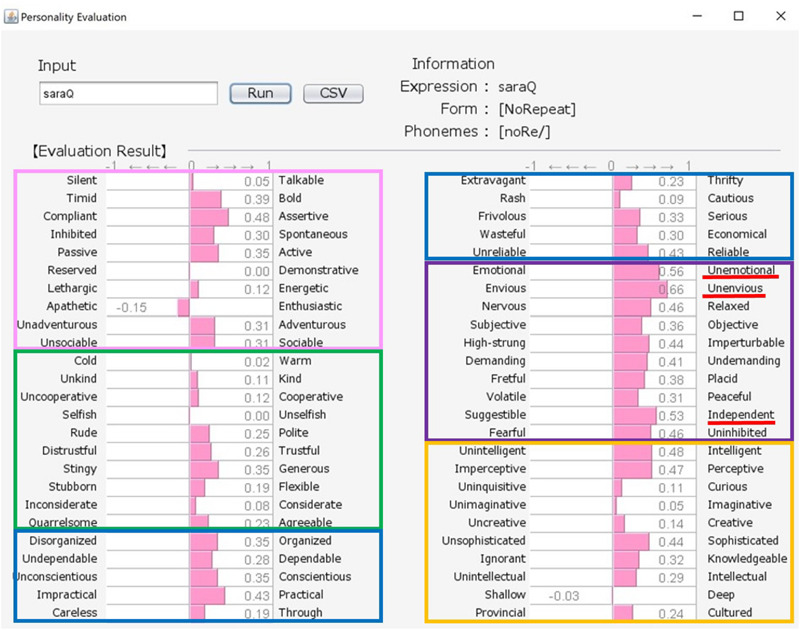
Output for “saraQ (nearly equal to frank)” personality. The pink, green, blue, purple, and orange frames indicate Extroversion, Agreeableness, Conscientiousness, Neuroticism, and Openness, respectively.

**FIGURE 10:**
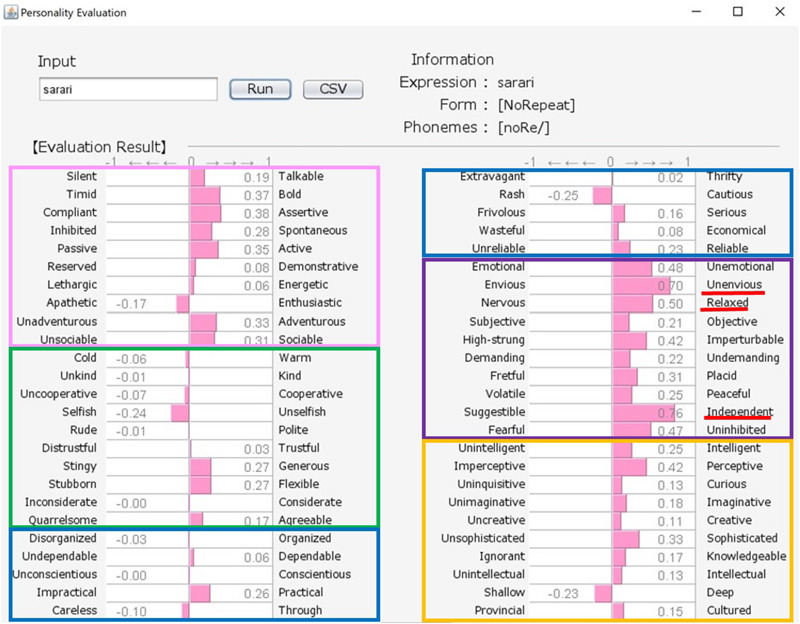
Output for “sarari (nearly equal to frank)” personality. The pink, green, blue, purple, and orange frames indicate Extroversion, Agreeableness, Conscientiousness, Neuroticism, and Openness, respectively.

**FIGURE 11:**
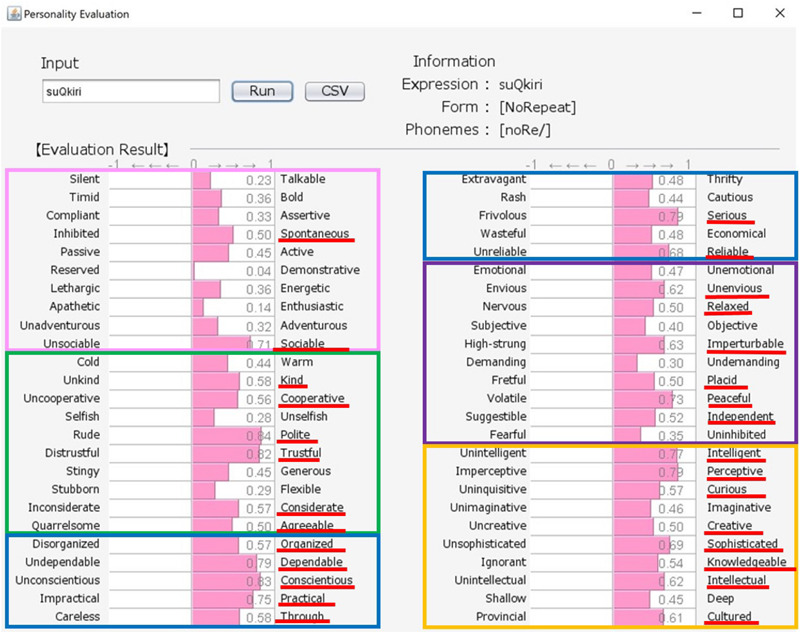
Output for “sukkiri (smart)” personality. The pink, green, blue, purple, and orange frames indicate Extroversion, Agreeableness, Conscientiousness, Neuroticism, and Openness, respectively.

Japanese people frequently use SSWs to express personality, such as “howa-howa,” which indicates a soft but unreliable person. SSWs are used in everyday conversation and a single SSW tends to convey more information than one adjective, as shown in the system output example for “howa-howa” in Figure 1. Japanese has a large SSW vocabulary that can express complex details about personality. At the same time, meanings of SSWs are typically characterized by phonetic features associated with multiple sensory experiences (e.g., Sakamoto and Watanabe, 2016 for taste; Sakamoto and Watanabe, 2018 for touch). In addition, Kawahara et al. (2015) showed a direct relationship between sensory impression (visual shape) and personality. We tested the relationship between tactile impression and personality using a Google search. For 60 SSWs given in Komatsu et al. (2012), we used the search terms “SSW person” (for personality expression) and “SSW touch” (for tactile expression) in a Google search query on May 7, 2020. More than 10,000 search results were obtained for both personality and tactile texture for the following 8 SSWs: “hunwari” and “huwa-huwa” (SSWs related to softness); “shikkari” (related to hardness); and “sappari,” “assari,” “saraQ,” “sarari,” and “sukkiri” (related to dryness). Although these expressions are all used for both tactile aspects and personality, they behave differently. “Hunwari” and “huwa-huwa” are both related to softness of touch. However, as shown in Figures 4, 5, the “hunwari” personality (nearly equal to gentle personality) is warm, kind, flexible, and relaxed, while the “huwa-huwa personality (nearly equal to fluffy personality) is unreliable. The “hunwari” personality seems more positive. The meanings of “shikkari” in terms of touch and personality are likely to share commonalities. “Shikkari” is related to hardness of touch, while the “shikari” personality (solid personality) is, as shown in Figure 6, polite, trustful, conscientious, serious, and intelligent. “Sappari,” “assari,” “saraQ,” “sarari,” and “sukkiri” are all related to dryness of touch, while their meanings for personality are different but nearly equal to frank, as shown in Figures 7–11.

A limitation of our system is that it is available only in Japanese, because the application was constructed using Japanese SSWs. However, it has been argued that sensory-sound associations for auditory, visual, tactile, and olfactory perceptions are universal phenomena observed in many languages, especially Asian-African languages (Dingemanse, 2012). Sound-symbolic features also occur in Indo-European languages, such as English (Bloomfield, 1933; Bolinger, 1950; Crystal, 1995). For example, roughly half of the English words starting with “gl-” (such as, glance, glare, glass, glimpse, and glow) imply something visual and bright (Crystal, 1995). Studies have found world-wide sound symbolism in words referring to visual shapes, such as “mal” vs. “mil” (Sapir, 1929) and “bouba” vs. “kiki” (Ramachandran and Hubbard, 2001) for round vs. sharp shapes.

We believe that sensory-sound associations for personality description have been overlooked, although there are languages that use SSWs to express personality. For example, the SSW “lumbud-lumbud” in Mundari (the South Asian linguistic area), whose original meaning is the appearance of a hole opening and closing, can also refer to a person that cannot keep a secret and frequently shares information inappropriately with others (Badenoch et al., 2019). The SSW “gusu-gusu” describes a slow and silent, inactive personality (Osada et al., 2019). In addition, sound-symbolic features related to personality traits have been found in Indo-European languages, such as English and Spanish (Lindauer, 1990; Milán et al., 2013; Kawahara et al., 2015). It would be interesting to investigate the differences in personality categories among different languages, as our approach might be applicable to other languages that have SSWs. Future research could explore the reasons why some SSWs represent personality, and why these are particularly highly developed in Japanese.

## Conclusion

In this paper, we focused on SSWs that can express complex aspects of personality traits, and constructed a system that can convert a SSW into values in terms of 50 personality-related adjective pairs. This system can obtain information equivalent to the adjective scales using only a single word instead of asking many questions. The prediction accuracy of the system was tested by calculating the multiple correlation coefficients, and it was found that those of 48 personality-related adjective pairs exceeded 0.75. An evaluation experiment, in which participants rated the appropriateness of the system output, was also performed, and the result demonstrated the effectiveness of the system. We believe that this system can contribute to the field of personality computing.

## Data Availability Statement

The original contributions presented in the study are included in the article, further inquiries can be directed to the corresponding author.

## Ethics Statement

This study was reviewed and approved by the ethics committee of The University of Electro-Communications, Tokyo, Japan. The patients/participants provided their written informed consent to participate in this study.

## Author Contributions

MS and JW conceived the experiments. MS performed the experiment. JW and KY carried out the data analyses. All authors discussed and interpreted the results, and contributed to drafts of this manuscript.

## Conflict of Interest

JW is employed by the NTT Communication Science Laboratories, Nippon Telegraph and Telephone Corporation as a research scientist conducting basic scientific research on human sensory processing. There are no patents, products in development, or marketed products to declare. This does not alter the authors’ adherence to the journal’s policies on sharing data and materials. The remaining authors declare that the research was conducted in the absence of any commercial or financial relationships that could be construed as a potential conflict of interest.
